# Endoscopic management versus radical nephroureterectomy for localized upper tract urothelial carcinoma in a high endemic region

**DOI:** 10.1038/s41598-021-83495-4

**Published:** 2021-02-17

**Authors:** Yung-Tai Chen, Chih-Chin Yu, Hsin-Chih Yeh, Hsiang-Ying Lee, Yuan-Hong Jiang, Yu-Khun Lee, Chia-Hao Kuei, Chia-Chang Wu, Chao-Yuan Huang, Wei-Yu Lin, Cheng Kuang Yang, Yao Chou Tsai

**Affiliations:** 1 Department of Urology, Postal Hospital, Taipei, Taiwan; 2 Department of Urology, Taiwan Adventist Hospital, Taipei, Taiwan; 3 Division of Urology, Department of Surgery, Taipei Tzuchi Hospital, The Buddhist Tzu Chi Medical Foundation, New Taipei City, Taiwan; 4 School of Medicine, Buddhist Tzu Chi University, Hualien, Taiwan; 5 Department of Urology, Kaohsiung Municipal Ta-Tung Hospital, Kaohsiung, Taiwan; 6 Department of Urology, Kaohsiung Medical University Hospital, Kaohsiung, Taiwan; 7 Department of Urology, School of Medicine, College of Medicine, Kaohsiung Medical University, Kaohsiung, Taiwan; 8 Department of Urology, Hualien Tzu Chi Hospital, Buddhist Tzu Chi Medical Foundation and Tzu Chi University, Hualien, Taiwan; 9 Division of Urology, Department of Surgery, Cardinal Tien Hospital, New Taipei City, Taiwan; 10 Department of Urology, School of Medicine, Fu-Jen Catholic University, Taipei, Taiwan; 11 Department of Urology, Shuang Ho Hospital, Taipei Medical University, New Taipei City, Taiwan; 12 Department of Urology, School of Medicine, College of Medicine, Taipei Medical University, Taipei, Taiwan; 13 Department of Urology, National Taiwan University Hospital, College of Medicine, National Taiwan University, Taipei, Taiwan; 14 Division of Urology, Department of Surgery, Chang Gung Memorial Hospital, Chia-Yi, Puzi City, Taiwan; 15 Chang Gung University of Science and Technology, Chia-Yi, Puzi City, Taiwan; 16 Department of Medicine, Chang Gung University, Taoyuan, Taiwan; 17 Department of Urology, Taipei Medical University Hospital, Taipei Medical University, No. 252, Wuxing St., Xinyi District, Taipei City, 110 Taiwan

**Keywords:** Ureter, Urology

## Abstract

Our aim was to analyze the clinical and survival differences among patients who underwent the two main treatment modalities, endoscopic ablation and radical nephroureterectomy. This study examined all patients who had undergone endoscopic management and RNU between Jul. 1988 and Mar. 2019 from the Taiwan UTUC registry. The inclusion criteria were low stage UTUC in RNU and all cases in endoscopic managed UTUC with a curative intent. The demographic and clinical characteristics were included for analysis. In total, 84 cases in the endoscopic group and 272 cases in the RNU group were enrolled for final analysis. The median follow-up period were 33.5 and 42.0 months in endoscopic and RNU group, respectively (*p* = 0.082). Comparison of Kaplan–Meier estimated survival curves between groups, the endoscopic group was associated with similar overall survival (OS), cancer specific survival (CSS), and intravesical recurrence free survival (IVRS) but demonstrated inferior disease free survival (DFS) (*p* = 0.188 for OS, *p* = 0.493 for CSS and *p* < 0.001 for DFS). Endoscopic management of UTUC was as safe as RNU in UTUC endemic region.

## Introduction

Upper tract urothelial carcinoma (UTUC) is an uncommon cancer which accounts for 5–10% of genitourinary urothelial cancers (UC) in Western countries. In a UTUC-endemic area like Taiwan, it accounts for an unusually high incidence (20–30%) for all UC^1–5^. In addition to the highly endemic nature, an unusually high female prevalence of UTUC in Taiwan also revealed unique features when compared with UTUC in other regions worldwide^6,7^. These unique characteristics might imply a different behavior for UTUC in Taiwan. For the above reasons, we set up a nation-wide multi-institution UTUC database containing detailed clinical, histological and treatment information in order to reveal the hidden risks which lead to the high endemic and predominantly female UTUC in Taiwan.

Radical nephroureterectomy (RNU) with bladder cuff excision is the gold standard for treatment of UTUC. However, in cases with a solitary kidney, suboptimal renal function or cases of major medical co-morbidities, RNU may not be a feasible treatment option. Kidney sparing surgery (KSS) like retrograde intra-renal endoscopic ablation, percutaneous endoscopic ablation or segmental resection of UTUC has become a primary treatment option for those patients with low risk tumors. The outcomes and clinical benefits of KSS for UTUC have been explored for more than two decades in Western countries^8–14^. However, the clinical benefit of KSS were rarely explored in the Asian UTUC cohort, not even in high endemics region like Taiwan^15,16^. This prompted us to analyze the clinical and survival differences among patients who underwent the two main treatment modalities, endoscopic ablation and RNU, in Taiwan.

## Material and methods

The Taiwan UTUC (upper tract urothelial carcinoma) registry is a multicenter, internet-based UTUC registry in which 95 participating surgeons from 12 different hospitals in Taiwan registered their patients who underwent UTUC treatments. This study was approved by the institutional review board of Taipei Tzu Chi General Hospital (IRB no.:06-X34-105) and the study protocols and methods were carried out in accordance with relevant guidelines and regulations. The informed consent was also obtained from all participants. This study examined the retrospective data of all patients who had undergone endoscopic management and RNU between Jul. 1988 and Mar. 2019. The clinical staging system used in endoscopic groups was based on biopsy histology (or washing cytology: optional if failed biopsy), and cross-sectional imaging. The inclusion criteria were low stage UTUC in RNU and all cases in endoscopic managed UTUC with a curative intent. The definition of low stage UTUC in RNU group is a pT stage not higher than pT2 and a negative nodal/metastasis status based on imaging or final histological analysis. The exclusion criteria were clinical/pathological T3, T4, node positive disease, those who initially received endoscopic management then shifted to RNU and who were lost to follow-up with unknown disease status.

**Figure 1:**
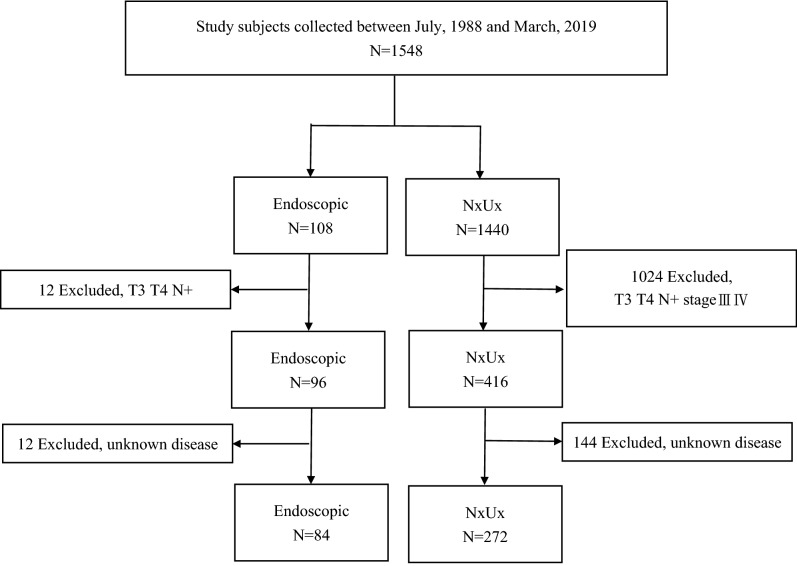
Flow diagram of case recruitment process.

In total, 1548 patients were examined for eligibility, with 356 cases enrolled for final analysis (Fig. 1). The groups were categorized by endoscopic or RNU management of UTUC. The demographic and clinical characteristics included for analysis were age, gender, ECOG, BMI, comorbidities, risk factors, clinical tumor information, histologic findings, creatinine levels, status of local/systemic adjuvant treatment, post-operative complication, and disease survial/free status. The tumor staging was according to AJCC (American Joint Committee on Cancer) TNM staging system and histologic grade was according to WHO/ISUP recommendation grading system. Cross-sectional imaging was used to determine recurrence/progression-free status in RNU cases. Ureteroscopy examination is the standard of follow-up in each endoscopic managed case, but those with suspicious urinary tract local recurrence were given additional cross-sectional imaging to rule out the possibility of locally advanced or metastatic disease. In addition, those endoscopic cases that could not be successfully biopsied were confirmed with urothelial cancer by washing cytology (three out of 84 cases).

Differences between groups were compared using two-sample t-test for continuous variables, Pearson Chi-square for categorical variables. Continuous variables here tested for normality using Kolmogorov–Smirnov test. The Kaplan–Meier estimator was used to estimate the rates of prognostic outcomes, and the survival curves were compared using the stratified log-rank test. Cox proportional hazard model was selected to assess the effect of the surgical approaches on the prognostic outcomes, alone and after adjusting for potential confounders. All statistical assessments were two-tailed and considered statistically significant at p < 0.05. Statistical analyses were carried out with IBM SPSS statistical software version 22. (The description of statistical methods was based on standard format of statistical analysis of Taiwan UTUC collaboration group).

## Results

### Baseline characteristics

Table 1 summarizes the baseline clinical characteristics of the 84 cases in the endoscopic group and 272 cases in the RNU group. Of the 84 cases two had bilateral renal units managed endocopically and of the 272 cases four had bilateral renal units managed by RNU. These two groups were comparable among most baseline characteristics except performance status, history of tobacco use, pre-operative end stage renal disease (ESRD) and previous history of RNU. Patients in the endoscopic group had more previous contralateral RNU and fewer cases with pre-operative ESRD when compared with the RNU group. The endoscopic group had more renal pelvis tumors and tumor muti-focality when compared with the RNU group. In addition, the RNU group was associated with more larger tumor (> 2 cm) when compared with the endoscopic group. Both groups had comparable tumor characteristics in laterality of kidney affected, pre-operative cytology status, and pre-operative histological results according to biopsy. The patients in the endoscopic group were associated with more previous bladder urothelial cancer.

**Table 1:** Baseline demographic data of endoscopic and nephroureterectomy (RNU) managed UTUC.

Variables	Endoscopic		RNU		p-value^a^
	N	%	N	%	
**Gender**					
Men	36	(42.90)	118	(43.40)	0.932
Women	48	(57.10)	154	(56.60)	
Age^b^ mean ± SD	68.8 ± 11.5		67.6 ± 10.6		0.331
BMI^b^ mean ± SD	23.7 ± 3.8		24.1 ± 3.8		0.431
Hospital stay (day)^b^ median	2.0		8.0		< 0.001**
Follow up (months)^b^ median	33.5		42.0		0.082
**ECOG**					
Normal activity fully ambulatory	64	(76.19)	125	(45.96)	0.002**
Symptoms, but nearly fully ambulatory	17	(20.24)	101	(37.13)	
Some bed time, but needs to be in bed less than 50% of normal daytime	3	(3.57)	10	(3.68)	
Needs to be in bed more than 50% of normal daytime	0	(0.00)	1	(0.37)	
**Risk factor**					
Smoking	4	(4.76)	51	(18.75)	0.016*
Chemical exposure	0	(0.00)	14	(5.15)	0.068
Herbal supplements	3	(3.57)	32	(11.76)	0.106
Arsenic water	1	(1.19)	17	(6.25)	0.145
ESRD/CRI	18	(21.43)	60	(22.06)	0.193
Previous nephroureterectomy for UC	18	(21.43)	7	(2.57)	< 0.001**
Regular hair coloring	2	(2.38)	25	(4.75)	0.119
**Comorbidity**					
CAD	8	(9.52)	31	(11.40)	0.604
Arrythmi	4	(4.76)	10	(3.68)	0.672
HTN	49	(58.33)	134	(49.26)	0.177
ESRD	4	(4.76)	39	(14.34)	0.017*
DM	23	(27.38)	70	(25.74)	0.816
Gout	9	(10.71)	12	(4.41)	0.035*
GI	9	(10.71)	33	(12.13)	0.693
Malignancy (No UTUC/bladder UC)	7	(8.33)	30	(11.03)	0.457
**Tumor location**					
Renal pelvis	73	(86.90)	172	(63.24)	< 0.001**
Ureter	22	(26.19)	141	(51.84)	< 0.001**
**Tumor size (cm)**					
< 2	74	(88.10)	102	(37.50)	< 0.001**
≥ 2	7	(8.33)	163	(59.93)	
**Affected kidney at diagnosis**					
Left	41	(48.81)	123	(45.22)	0.253
Right	40	(47.62)	144	(52.94)	
Bilateral	2	(2.38)	4	(1.47)	
**Multifocality**					
Not available	2	(2.38)	1	(0.37)	< 0.001**
No	16	(19.05)	191	(70.22)	
Yes	62	(73.81)	77	(28.31)	
**Pre-op urine cytology**					
Negative	18	(21.4)	36	(13.2)	0.658
Atypia	18	(21.4)	48	(17.6)	
Positive	33	(39.3)	90	(33.1)	
**Synchronous bladder urothelial cancer**					
Previous Hx of bladder UC	20	(23.81)	29	(10.66)	0.001**
Concurrent bladder UC	7	(8.33)	41	(15.07)	
Preoperative hydronephrosis	47	(55.95)	155	(56.99)	0.767
Bladder UC after RNU/endoscopic ablation	19	(22.62)	91	(33.46)	0.481
Definitive chemotherapy for advanced/metastasis UTUC	7	(8.33)	41	(15.07)	0.178
Radiation therapy for UTUC	2	(2.38)	14	(5.15)	0.251
**Biopsy tumor grading**					
Benign lesion	0	(0.00)	5	(1.84)	0.317
Papillary urothelial neoplasm of low malignant potential	0	(0.00)	4	(1.47)	
Low grade	28	(33.33)	60	(22.06)	
High grade	43	(51.19)	84	(30.88)	
G2 (WHO 1973)	0	(0.00)	5	(1.84)	
Dysplasia/atypia	2	(2.38)	6	(2.21)	
Biopsy failure	3	(3.57)	8	(2.94)	
**Clinical T stage T**					
cTx	72	(85.71)	196	(72.06)	0.008**
cTa	7	(8.33)	15	(5.51)	
cT1	3	(3.57)	41	(15.07)	
cT2	2	(2.38)	20	(7.35)	

^a^Chi-Squared test calculated for the difference Variables.

^b^Student’s t-test calculated for the difference in means.

*< 0.05.

**< 0.01.

### Treatment outcomes and kidney function

The endoscopic group had a shorter mean hospital stay (2 versus 8 days, *p* < 0.001) (Table 1). The RNU group was associated with more Clavien-Dindo grade II and higher grade of complications than the endoscopic group (OR (95% CI) 7.445 (2.630–21.07), *p* < 0.001) (Table 2). Endoscopic managed UTUC was commonly (25%) associated with ureteral strictures. The risk of progression into ESRD in pre-treatment non-ESRD cases after intervention were comparable between groups (OR (95% CI) 0.745 (0.422–1.315); *p* = 0.31). There was no significant difference in pre-operative and post-operative case number changes in normal renal function, chronic kidney disease (CKD) or ESRD status between groups (Table 2). Both groups also had comparable mean pre- and post-operative creatinine and eGFR level during all periods of follow-up. However, the Endoscopic group was significantly associated with less eGFR decrease than the RNU group (8.24 versus 13.37, *p* = 0.032).

**Table 2:** Clinical outcome data of endoscopic and nephroureterectomy (RNU) managed UTUC patients.

Variables	Endoscopic		RNU		p-value^a^
	N	%	N	%	
Pre-OP Cr level (mg/dl)^b^ mean ± SD	2.11 ± 1.9		1.33 ± 2.82		0.898
Pre-OP platelet (× 10^3^/μl)^b^ mean ± SD	206.3 ± 56.5		210.0 ± 76		0.209
Pre-OP Hgb^b^ mean ± SD	11.82 ± 1.98		11.80 ± 2.06		0.642
Post-OP (final) Cr level (mg/dl)^b^ mean ± SD	3.34 ± 3.01		1.80 ± 2.73		0.736
Post-OP 1 month Cr level (mg/dl)^b^ mean ± SD	3.57 ± 10.5		1.61 ± 2.49		0.381
**Surgical complications (Clavien–Dindo classification)**					
Grade I	12	(14.29)	49	(18.01)	
OR (95% Cl)	3.208 (1.786, 5.763)				0.386
Grade II	4	(4.76)	72	(26.47)	
OR (95% Cl)	7.445 (2.630, 21.07)				< 0.001**
Grade III	4	(4.76)	9	(3.31)	
OR (95% Cl)	0.700 (0.210, 2.334)				0.561
Grade IV	0	(0.00)	10	(3.68)	0.071
Grade V	0	(0.00)	1	(0.37)	0.574
**Post-OP complication**					
ESRD	24	(28.6)	74	(27.2)	
OR (95% Cl)	0.745 (0.422, 1.315)				0.310
Ureter stricture	21	(25.0)	0	(0.00)	< 0.001**
**Mortality**					
No	53	(63.10)	154	(56.62)	0.003**
UTUC related	6	(7.14)	21	(7.72)	
Non-UTUC related	2	(2.38)	50	(18.38)	
**Disease free**					
No	47	(55.95)	48	(17.65)	< 0.001**
Yes	37	(44.05)	224	(82.35)	

^a^Chi-Squared test calculated for the difference Variables.

^b^Student’s t-test calculated for the difference in means.

*< 0.05.

**< 0.

### Survival analysis

**Figure 2:**
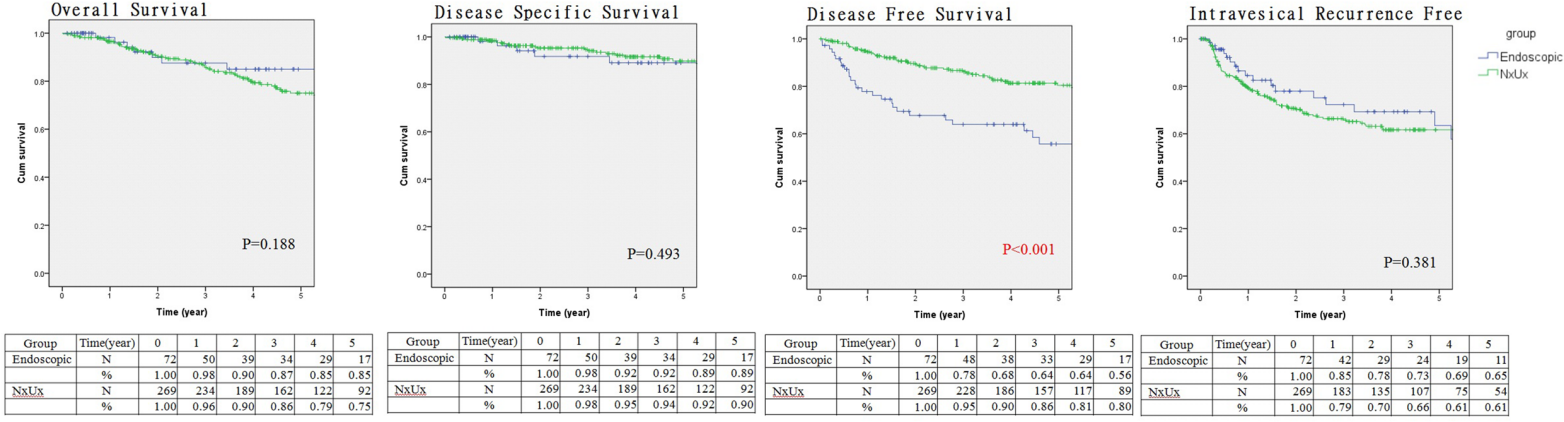
Kaplan–Meier survival curves of overall survival, cancer specific survival, disease free survival and intravesical recurrence free survival stratified by endoscopic or nephroureterectomy management (survival curves were created and analyzed by SPSS software version 26).

**Figure 3:**
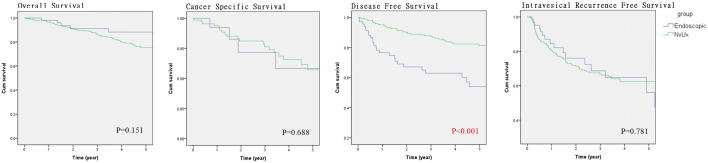
Kaplan–Meier survival curves of overall survival (OS), cancer specific survival, disease free survival (CSS) and intravesical recurrence free survival (IVRFS) stratified by endoscopic or nephroureterectomy management after adjustment of confounding factors. (Tumor location, multi-focality, and concurrent bladder urothelial cancer) (survival curves were created and analyzed by SPSS software version 26).

The median follow-up period were 33.5 and 42.0 months for the endoscopic and RNU group, respectively (*p* = 0.082). Comparison of Kaplan–Meier estimated survival curves between groups, showed the endoscopic group was associated with similar overall survival (OS), cancer specific survival (CSS), and intravesical recurrence free survival (IVRS) but demonstrated inferior disease free survival (DFS) (*p* = 0.188 for OS, *p* = 0.493 for CSS and *p* < 0.001 for DFS) (Fig. 2). On multivariable Cox regression analyses that controlled for the potential clinical and pathological confounding factors, the RNU group was associated with significantly better DFS than the endoscopic group (hazard ratio 0.078, 95% confidence interval 0.018–0.336 for DFS; p = 0.001), but endoscopic treatment did not affect the outcome in OS, CSS, IVRS) (Supplementary Tables 2, 3, 4, 5). Although the RNU group was associated with more large (> 2 cm) tumors which might have an impact on disease survival, however, the discrepancies of disease volume among groups did not affect the survival outcomes in all survival domains based on the multi-variance analysis (Supplement Tables 2, 3, 4, 5). In survival curve analysis controlling for the potential clinical and pathological confounding variables (history of tobacco use, prior RNU for UTUC, ECOG, and muli-focality), the endoscopic group was comparable to the RNU group in the OS, CSS, and IVRS, but showed an inferior DFS in the endoscopic group (p < 0.001) (Fig. 3).

## Discussion

To the best of our knowledge, this is the first real world like comprehensive comparative study using a well-maintained nation-wide registry with detailed clinical, histological, and outcome data in upper tract urothelial cancer. So far, there are only two comparative trials which compared nephron-sparing surgery to RNU for UTUC both essentially based on the Surveillance, Epidemiology, and End Results program (SEER) database that is predominantly Caucasian^8,17^. Although these two studies were larger comparative cohorts, they were limited by lack of clinical, histological, and detailed outcome information, making a deeper multivariate analysis infeasible. The current study is the first comparative and largest cohort study among Asian populations, and hence, could fill a knowledge gap in the literature. Based on our results, there were no significant survival differences between groups in terms of OS, CSS and intra-vesical recurrence free survival in this nation-wide study. Although our study enrolled more high grade tumors, this real world data was comparable to the systemic review reported by Seisen T, et al., which revealed no significant difference in CSS between RNU and KSS, although KSS was associated with inferior recurrence free survival than RNU^16^. Current results were also comparable to previously mentioned SEER database studies^8,17^. Therefore, endoscopic management of high grade predominant localized low stage UTUC was as safe and effective as RNU in cancer control in a high endemic region.

The differences from other historical cohorts are the enrollment of a mainly Asian population, predominant high grade tumors (50%) and low clinical T/N0 stage based on pre-operative cross sectional image in the endoscopic cohort. There were several reasons that we enrolled high grade and low stage UTUC: (1) grade migration is common after RNU, therefore, biopsy confirmed low grade tumor could be high grade in reality. (2) Differentiation between cT1 and cT2 stage is clinically not feasible based on biopsy and image results and (3) high grade tumor is more predominant (more than 70%) in Taiwan population, therefore, excluding high grade tumors usually excludes kidney-sparing option for most patients with imperative indications in Taiwan. In our cohort, it accounted for 28/43 of all endoscopic group and 11/28 of them were free of disease after endoscopic treatment. In short, enrollment of high grade and low stage (cT1-2) UTUC is a more real-world like comparison between endoscopic treatment and RNU.

With basically similar baseline clinical and histological characteristics among groups, the endoscopic group was associated with inferior DFS in multivariate survival analysis and survival curve analysis with confounding factor adjustment. UTUC is usually associated with high multi-focality (> 70% in endoscopic group and > 1/3 in Taiwan UTUC registry) and upper tract recurrence, therefore, re-ablation is very common for endoscopically managed patients. In our endoscopic group, each patient received a median of two endoscopic ablations and the number of ablations ranged from 1 to 13. In a long-term follow-up cohort reported by Cutress, a recurrence occurred in 68% of all endoscopically treated UTUC at a median follow-up of 4.5 years^12^. Our endoscopic cohort also mirrors a high incidence of recurrence which was found in 64% of all endoscopically managed UTUC after 5 years of follow-up. In addition, the recurrence rate actually increased with time according to our recurrence free survival curve analysis (Figs. 2, 3). Therefore, this evidence stress the importance of regular long-term follow-up with a stringent protocol and a careful case selection criteria for KSS in UTUC.

The clinical benefits of nephron-sparing surgery (NSS) for renal cancer have been well explored in the last decade. In a recent meta-analysis that explored the incident of CKD after radical nephrectomy (RN) or NSS, it concluded that NSS was associated with significant reduction in the incidence of stage 3 or higher CKD and better survival^18^. Although KSS for UTUC has been developed to preserve more renal units for more than two decades, the benefit of renal function preservation and risk of progression into ESRD in the long-run had rarely been described. In our median 3 years follow-up, 45% of endoscopically treated patients had normal renal function (eGFR > 60 ml/min per 1.73 m^2^) before treatment, but only half of them remained normal at the end of follow-up and the results were comparable to those in the RNU group (Table 2). At the end of our follow-up, 38% and 34% of newly developed ESRD were identified in endoscopic and RNU group, respectively, and the difference among groups were not significant. In addition, there were no significant differences in pre-operative and post-operative (1 month and at last follow-up) creatinine/eGFR levels among groups. The only advantage in the endoscopic group was a smaller eGFR decline (− 8.24 versus − 13.37, *p* = 0.032) at the end of follow-up when compared with RNU cohort. Although, endoscopic management for UTUC actually preserved more renal renal units post-operatively, it only delayed the development of ESRD but did not eliminate the risk of hemodialysis in the long-run.

**Figure 4:**
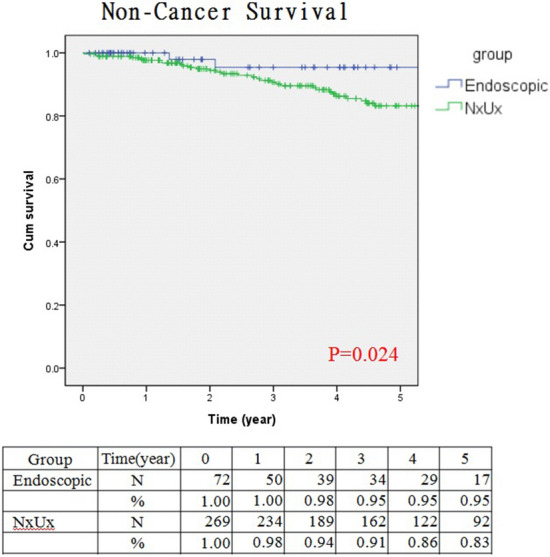
Kaplan–Meier survival curves of non-cancer survival stratified by endoscopic or nephroureterectomy management. (Survival curves were created and analyzed by SPSS software version 26).

It has been confirmed that NSS for renal cancer was associated with a lower risk of non-renal cancer related mortality and overall mortality, although whether KSS of UTUC is associated with lower risk of non-cancer (other cause) related mortality remains scarce in the literature^18^. In a population-based study published in 2014, which compared localized non-invasive UTUC being treated with either KSS or RNU, they found similar CSS among groups^17^. However, the KSS cohort was associated with more non-UTUC (other cause) related mortality. This finding is contrary to the evidence extracted from NSS for renal cancer and also our observations of the Taiwan UTUC registry. In our study, we basically had similar general healthy status (co-morbidities and risk factors) among groups, which were important baseline cohort characteristics and would potentially affect the non-UTUC related mortality and actually not reachable in most published registry studies. We found that the endoscopically treated cohort was associated with lower non-UTUC related mortality (3.3% versus 22%, p = 0.003), but comparable UTUC related mortality (9.8% versus 9.3%) among groups. The above findings were also confirmed on our 5-year non-cancer (other cause) survival curve analysis which revealed better survival in the endoscopic cohort (Fig. 4; *p* = 0.024). We argue that RNU is possibly associated with inferior non-UTUC (other cause) related survival due to early loss of renal units immediately after RNU, therefore, a prospective randomized study is mandatory to confirm this speculation in the near future.

Prior diagnostic ureteroscopy has been reported to be associated with more intravesical recurrence after RNU^19,20^. However, two meta-analyses focusing on the factors associated with intravesical recurrence revealed that patient, tumor and treatment specific factors are the risks for recurrence but prior diagnostic ureteroscopy is not^21,22^. Diagnostic ureteroscopy is commonly the main follow-up strategy for UTUC following KSS. In our nation-wide cohort, most of the endoscopically managed UTUC were followed with ureteroscopy. It means that our endoscopic group experienced regular, repeated ureteroscopy studies following endoscopic ablation whether the urothelial cancer was clear or not. Interestingly, we did not find an increased risk of intravesical recurrence in the endoscopic cohort. In addition, after multivariate analysis with adjustment for confounding factors, prior history of bladder UC and concurrent bladder UC were independent risks for intravesical recurrence for all UTUC. A large cohort study focusing on the impact of diagnostic ureteroscopy after RNU also found that bladder UC was the main risk for intravesical recurrence but not diagnostic urteroscopy^23^. To date, the pathophysiology or mechanism of lower tract seeding of urothelial cancer still remains unclear and most of the published evidences that favored diagnostic ureteroscopy as a risk factor were biased due to not excluding cases with prior bladder UC^23^. In brief, it is still controversial as to whether diagnostic ureteroscopy is a major reason increasing the risk of intravesical recurrence, and a well designed randomized trial is mandatory to answer this unsolved issue.

### Limitation

This study was limited by its retrospective design, however, the multi-institution enrollment and multivariate Cox regression analyses plus confounding factor adjustment can minimize this bias. Missing data and data inconsistency are common limitations for clinical registry studies which were mainly managed by regular data auditing and panel consensus meeting by Taiwan UTUC collaboration group. In addition, lack of an independent dedicated or central pathological and imaging review might lead to varied histological and imaging reporting bias among institutes. To minimize the impact of in-concordance of imaging and pathology between different centers, we used a standardized histological and imaging report format which was approved by Taiwan Radiological and Pathology Society based on the AJCC TNM staging system and the principles of pathology management for urothelial cancer in NCCN guidelines to ensure a standardized pathology management protocol, therefore, minimizing the inter-observer staging bias. Finally, the limited endoscopic treatment cohort and most cohort patients being managed by a few experienced surgeons could limit the generalizability to inexperienced surgeons. Therefore, a further large scale randomized comparative trial is mandatory to answer the unsolved questions in current registry-based study.

## Conclusion

Endoscopic management of UTUC achieved comparable the OS, CSS and intravesical recurrence free survival as RNU in high grade predominant, UTUC endemic cohort, but it was associated with inferior DFS. Therefore, regular long-term follow-up with a stringent protocol and careful case selection criteria is important for endoscopic ablation of UTUC. Although, endoscopic management of UTUC could preserve more renal units, it possibly delayed the development of ESRD but did not eliminate the risk of dialysis in the long-run. Interestingly, endoscopic management of UTUC was associated with less non-UTUC related mortality, which deserves a prospective randomized trial to confirm this argument.

## Supplementary Information

Supplementary Tables.
