# Supplementary material for: Endoscopic management versus radical nephroureterectomy for localized upper tract urothelial carcinoma in a high endemic region
Source: Sci Rep. 2021 Feb 17;11:4040. doi: 10.1038/s41598-021-83495-4 (PMC7889610; doi:10.1038/s41598-021-83495-4)
Supplement: Supplementary file 1 — Supplementary Tables. [file 41598_2021_83495_MOESM1_ESM.docx]

Table supplement 1: Univariate survival analysis of overall, cancer specific, disease free, and intravesical recurrence free survival.

| Univariate analysis | OS | | CSS | | DFS | | IVRFS | |
| --- | --- | --- | --- | --- | --- | --- | --- | --- |
|  | HR (95% CI) | p-value | HR(95% CI) | p-value | HR(95% CI) | p-value | HR(95% CI) | p-value |
| Sex |  |  |  |  |  |  |  |  |
| Male | 1 |  | 1 |  | 1 |  | 1 |  |
| Female | 0.850 (0.544, 1.328) | 0.475 | 0.824 (0.387, 1.757) | 0.616 | 0.851 (0.546, 1.328) | 0.478 | 0.590 (0.404, 0.862) | 0.006** |
| Surgical approach |  |  |  |  |  |  |  |  |
| Endoscopic | 1 |  | 1 |  | 1 |  | 1 |  |
| RNU | 1.631 (0.781, 3.406) | 0.193 | 0.728 (0.292, 1.813) | 0.495 | 0.296 (0.187, 0.467) | <0.001** | 1.260 (0.750, 2.117) | 0.382 |
| Age |  |  |  |  |  |  |  |  |
| < 70 | 1 |  | 1 |  | 1 |  | 1 |  |
| ≥ 70 | 1.395 (0.868, 2.243) | 0.169 | 0.907 (0.416, 1.980) | 0.807 | 1.261 (0.805, 1.975) | 0.310 | 1.176 (0.803, 1.722) | 0.405 |
| BMI |  |  |  |  |  |  |  |  |
| < 24 | 1 |  | 1 |  | 1 |  | 1 |  |
| ≥ 24 | 0.820 (0.498, 1.350) | 0.435 | 0.655 (0.286, 1.496) | 0.315 | 0.658 (0.404, 1.071) | 0.092 | 0.962 (0.644, 1.437) | 0.849 |
| ECOG |  |  |  |  |  |  |  |  |
| Normal activity fully ambulatory | 1 |  | 1 |  | 1 |  | 1 |  |
| Symptoms, but nearly fully ambulatory | 3.222 (1.852, 5.608) | <0.001** | 1.363 (0.569, 3.265) | 0.487 | 0.785 (0.455, 1.353) | 0.383 | 1.063 (0.695, 1.627) | 0.777 |
| Some bed time, but needs to be in bed less than 50% of normal daytime | 1.956 (0.584, 6.550) | 0.277 | 1.144 (0.149, 8.803) | 0.898 | 1.040 (0.323, 3.352) | 0.948 | 0 | 0.953 |
| Needs to be in bed more than 50% of normal daytime | 16.31 (2.140, 124.3) | 0.007** | 0 | 0.987 | 0 | 0.977 | 0 | 0.986 |
| Tumor location Renal pelvis |  |  |  |  |  |  |  |  |
| No | 1 |  | 1 |  | 1 |  | 1 |  |
| Yes | 0.760 (0.485, 1.190) | 0.230 | 1.052 (0.472, 2.345) | 0.901 | 1.409 (0.853, 2.326) | 0.180 | 0.824 (0.556, 1.222) | 0.336 |
| Tumor location Ureter |  |  |  |  |  |  |  |  |
| No | 1 |  | 1 |  | 1 |  | 1 |  |
| Yes | 1.646 (1.050, 2.579) | 0.030 | 1.715 (1.494, 3.704) | 0.169 | 0.965 (0.617, 0.310) | 0.877 | 1.859 (1.267, 2.726) | 0.002 |

(Continued to the neat page)

Table Continued

| Univariate analysis | OS | | CSS | | DFS | | IVRFS | |
| --- | --- | --- | --- | --- | --- | --- | --- | --- |
|  | HR (95% CI) | p-value | HR(95% CI) | p-value | HR(95% CI) | p-value | HR(95% CI) | p-value |
| Tumor size |  |  |  |  |  |  |  |  |
| < 2 cm | 1 |  | 1 |  | 1 |  | 1 |  |
| ≥ 2cm | 1.491 (0.934, 2.381) | 0.094 | 1.212 (1.450, 2.671) | 0.634 | 0.933 (0.593, 0.367) | 0.763 | 0.910 (0.620, 1.337) | 0.631 |
| Affected kidney at diagnosis |  |  |  |  |  |  |  |  |
| Left | 1 |  | 1 |  | 1 |  | 1 |  |
| Right | 1.241 (0.780, 1.974) | 0.362 | 0.970 (1.448, 2.097) | 0.937 | 0.749 (0.476, 3.379) | 0.212 | 1.501 (1.013, 2.224) | 0.043* |
| Bilateral | 5.798 (1.367, 24.59) | 0.017 | 5.189 (5.466, 40.42) | 0.116 | 3.296 (0.791, 0.373) | 0.101 | 0 | 0.963 |
| Multifocality |  |  |  |  |  |  |  |  |
| No | 1 |  | 1 |  | 1 |  | 1 |  |
| Yes | 1.323 (0.830, 2.108) | 0.240 | 2.439 (1.437, 5.233) | 0.022* | 2.873 (1.818, 2.342) | <0.001** | 1.582 (1.076, 2.327) | 0.020* |
| Pre-op Urine cytology |  |  |  |  |  |  |  |  |
| Negative | 1 |  | 1 |  | 1 |  | 1 |  |
| Atypia | 0.405 (0.127, 1.289) | 0.126 | 0.490 (0.489, 2.703) | 0.413 | 0.965 (0.415, 1.347) | 0.934 | 0.809 (0.399, 1.640) | 0.556 |
| positive | 1.005 (0.486, 2.075) | 0.990 | 1.334 (1.428, 4.157) | 0.619 | 1.352 (0.679, 9.391) | 0.391 | 1.101 (0.616, 1.968) | 0.746 |
| Synchronous bladder tumor |  |  |  |  |  |  |  |  |
| No | 1 |  | 1 |  | 1 |  | 1 |  |
| Previous Hx of bladder UC | 1.627 (0.874, 3.030) | 0.125 | 4.400 (1.418, 11.27) | 0.002** | 1.833 (1.035, 1.346) | 0.038* | 2.793 (1.774, 4.398) | <0.001** |
| Concurrent Bladder UC | 2.010 (1.159, 3.488) | 0.013* | 4.269 (2.480, 10.85) | 0.002** | 1.469 (0.793, 0.320) | 0.222 | 1.721 (1.026, 2.886) | 0.040* |
| Preoperative hydronephrosis |  |  |  |  |  |  |  |  |
| No | 1 |  | 1 |  | 1 |  | 1 |  |
| Yes | 1.182 (0.733, 1.907) | 0.492 | 3.511 (1.409, 10.20) | 0.021* | 1.983 (1.164, 4.379) | 0.012* | 1.146 (0.770, 1.707) | 0.502 |

(Continued to the neat page)

Table Continued

| Univariate analysis | OS | | CSS | | DFS | | IVRFS | |
| --- | --- | --- | --- | --- | --- | --- | --- | --- |
|  | HR (95% CI) | p-value | HR(95% CI) | p-value | HR(95% CI) | p-value | HR(95% CI) | p-value |
| clinical stage T |  |  |  |  |  |  |  |  |
| cTx | 1 |  | 1 |  | 1 |  | 1 |  |
| cTa | 1.135 (0.454, 2.835) | 0.786 | 0.514 (1.469, 3.807) | 0.515 | 0.376 (0.092, 7.336) | 0.173 | 1.076 (0.521, 2.223) | 0.842 |
| cT1 | 0.981 (0.499, 1.926) | 0.954 | 0.558 (0.432, 2.363) | 0.428 | 0.523 (0.210, 9.398) | 0.162 | 0.664 (0.344, 1.282) | 0.222 |
| cT2 | 0.226 (0.031, 1.633) | 0.141 | 0 | 0.981 | 1.202 (0.520, 1.380) | 0.667 | 0.508 (0.186, 1.386) | 0.186 |
| Smoking |  |  |  |  |  |  |  |  |
| No | 1 |  | 1 |  | 1 |  | 1 |  |
| Yes | 0.764 (0.397, 1.468) | 0.419 | 1.194 (0.430, 3.319) | 0.733 | 0.771 (0.374, 4.390) | 0.481 | 1.153 (0.687, 1.934) | 0.589 |
| Previous Nephroureterectomy for UC |  |  |  |  |  |  |  |  |
| No | 1 |  | 1 |  | 1 |  | 1 |  |
| Yes | 0.701 (0.253, 1.938) | 0.493 | 2.818 (0.426, 8.577) | 0.068 | 2.613 (1.298, 4.360) | 0.007** | 0.822 (0.357, 1.895) | 0.646 |

(Continued to the neat page)

Table Continued

| Univariate analysis | OS | | CSS | | DFS | | IVRFS | |
| --- | --- | --- | --- | --- | --- | --- | --- | --- |
|  | HR (95% CI) | p-value | HR(95% CI) | p-value | HR(95% CI) | p-value | HR(95% CI) | p-value |
| Clavien-Dindo classification |  |  |  |  |  |  |  |  |
| No | 1 |  | 1 |  | 1 |  | 1 |  |
| Yes | 1.456 (0.918, 2.311) | 0.110 | 1.519 (1.408, 3.259) | 0.283 | 1.216 (0.766, 1.331) | 0.407 | 0.828 (0.551, 1.244) | 0.363 |
| GradeⅠ |  |  |  |  |  |  |  |  |
| No | 1 |  | 1 |  | 1 |  | 1 |  |
| Yes | 1.146 (0.629, 2.087) | 0.656 | 0.883 (1.405, 2.556) | 0.818 | 0.944 (0.509, 6.349) | 0.855 | 0.717 (0.408, 1.259) | 0.247 |
| GradeⅡ |  |  |  |  |  |  |  |  |
| No | 1 |  | 1 |  | 1 |  | 1 |  |
| Yes | 1.249 (0.743, 2.101) | 0.402 | 1.057 (1.426, 2.621) | 0.905 | 0.831 (0.465, 4.385) | 0.532 | 0.969 (0.605, 1.551) | 0.895 |
| GradeⅢ |  |  |  |  |  |  |  |  |
| No | 1 |  | 1 |  | 1 |  | 1 |  |
| Yes | 3.557 (1.536, 8.235) | 0.003** | 5.015 (3.495, 16.821) | 0.009** | 3.540 (1.617, 0.352) | 0.002** | 1.082 (0.398, 2.944) | 0.878 |
| GradeⅣ |  |  |  |  |  |  |  |  |
| No | 1 |  | 1 |  | 1 |  | 1 |  |
| Yes | 1.243 (0.391, 3.955) | 0.712 | 2.379 (1.462, 10.079) | 0.239 | 2.008 (0.731, 7.320) | 0.177 | 1.110 (0.408, 3.018) | 0.838 |
| Post-OP Complication |  |  |  |  |  |  |  |  |
| No | 1 |  | 1 |  | 1 |  | 1 |  |
| Yes | 0.640 (0.351, 1.165) | 0.144 | 1.149 (0.483, 2.738) | 0.753 | 1.124 (0.647, 1.350) | 0.678 | 0.979 (0.616, 1.556) | 0.928 |
| ESRD |  |  |  |  |  |  |  |  |
| No | 1 |  | 1 |  | 1 |  | 1 |  |
| Yes | 0.727 (0.382, 1.382) | 0.331 | 1.356 (0.442, 3.393) | 0.515 | 0.868 (0.442, 3.303) | 0.680 | 0.918 (0.539, 1.566) | 0.755 |
| Ureter stricture |  |  |  |  |  |  |  |  |
| No | 1 |  | 1 |  | 1 |  | 1 |  |
| Yes | 0.044 (0.001, 2.589) | 0.133 | 0.044 (0.400, 33.063) | 0.356 | 1.496 (0.682, 1.381) | 0.314 | 1.424 (0.718, 2.822) | 0.311 |

Cl, confidence; HR, hazard ratio; OS, overall survival; CSS, cancer-specific survival; DFS, disease-free survival; IVRFS,Intravesical recurrence free survival

* < 0.05, ** < 0.01

Table Supplement 2: Multivariate survival analysis of overall survival.

| Multivariable analysis | OS | | | |
| --- | --- | --- | --- | --- |
|  | HR | (95% CI) | | **P** |
| Surgical approach |  |  |  |  |
| Endoscopic | 1 |  |  |  |
| RNU | 0.899 | 0.200 | 4.028 | **0.889** |
| ECOG |  |  |  |  |
| Normal activity fully ambulatory | 1 |  |  |  |
| Symptoms, but nearly fully ambulatory | 1.594 | 0.554 | 4.585 | **0.387** |
| Some bed time, but needs to be in bed less than 50% of normal daytime | 2.633 | 0.270 | 25.668 | **0.405** |
| Needs to be in bed more than 50% of normal daytime | 8.010 | 0.594 | 108.042 | **0.117** |
| Tumor location Renal pelvis |  |  |  |  |
| No | 1 |  |  |  |
| Yes | 0.884 | 0.320 | 2.438 | **0.811** |
| Affected kidney at diagnosis |  |  |  |  |
| Left | 1 |  |  |  |
| Right | 0.547 | 0.203 | 1.476 | **0.234** |
| Bilateral | 2.467 | 0.239 | 25.475 | **0.448** |
| Multiplicity |  |  |  |  |
| No | 1 |  |  |  |
| Yes | 2.113 | 0.637 | 7.011 | **0.221** |
| Synchronous bladder tumor |  |  |  |  |
| No | 1 |  |  |  |
| Previous Hx of bladder UC | 2.448 | 0.704 | 8.512 | **0.159** |
| Concurrent Bladder UC | 2.958 | 0.776 | 11.272 | **0.112** |
| Clavien-Dindo classification Grade Ⅲ |  |  |  |  |
| No | 1 |  |  |  |
| Yes | 1.150 | 0.125 | 10.591 | **0.902** |
| Tumor size |  |  |  |  |
| < 2 cm | 1 |  |  |  |
| ≥ 2cm | 2.198 | 0.721 | 6.700 | **0.166** |

Cl, confidence; HR, hazard ratio; OS, overall survival; CSS, cancer-specific survival; DFS, disease-free survival; BRFS, Bladder Recurrence-free survival

* < 0.05, ** < 0.01

Table supplement 3: Multivariate survival analysis of cancer specific survival.

| Multivariable analysis | CSS | | | |
| --- | --- | --- | --- | --- |
|  | HR | (95% CI) | | **P** |
| Surgical approach |  |  |  |  |
| Endoscopic | 1 |  |  |  |
| RNU | 0.503 | 0.083 | 3.046 | **0.454** |
| Multiplicity |  |  |  |  |
| No | 1 |  |  |  |
| Yes | 3.982 | 0.796 | 19.930 | **0.093** |
| Synchronous bladder tumor |  |  |  |  |
| No | 1 |  |  |  |
| Previous Hx of bladder UC | 2.927 | 0.630 | 13.604 | **0.171** |
| Concurrent Bladder UC | 4.252 | 0.878 | 20.589 | **0.072** |
| Preoperative hydronephrosis |  |  |  |  |
| No | 1 |  |  |  |
| Yes | 1.954 | 0.212 | 17.987 | **0.554** |
| Clavien-Dindo classification Grade Ⅲ |  |  |  |  |
| No | 1 |  |  |  |
| Yes | 0.000 | 0.000 | - | **0.980** |
| Tumor size |  |  |  |  |
| < 2 cm | 1 |  |  |  |
| ≥ 2cm | 2.412 | 0.465 | 12.515 | **0.294** |

Cl, confidence; HR, hazard ratio; OS, overall survival; CSS, cancer-specific survival; DFS, disease-free survival; IVRFS, Intravesical recurrence free survival

* < 0.05, ** < 0.01

Table supplement 4: Multivariate survival analysis of disease free survival.

| Multivariable analysis | DFS | | | |
| --- | --- | --- | --- | --- |
|  | HR | (95% CI) | | **P** |
| Surgical approach |  |  |  |  |
| Endoscopic | 1 |  |  |  |
| RNU | 0.045 | 0.008 | 0.265 | **0.001** |
| Multiplicity |  |  |  |  |
| No | 1 |  |  |  |
| Yes | 1.321 | 0.338 | 5.163 | **0.689** |
| Synchronous bladder tumor |  |  |  |  |
| No | 1 |  |  |  |
| Previous Hx of bladder UC | 0.816 | 0.213 | 3.131 | **0.767** |
| Concurrent Bladder UC | 3.553 | 0.739 | 17.089 | **0.114** |
| Preoperative hydronephrosis |  |  |  |  |
| No | 1 |  |  |  |
| Yes | 0.663 | 0.173 | 2.536 | **0.548** |
| Post-OP urine cytology |  |  |  |  |
| Negative | 1 |  |  |  |
| Atypia | 9.083 | 1.829 | 45.114 | **0.007** |
| Positive | 4.326 | 1.138 | 16.441 | **0.032** |
| previous Nephroureterectomy for UC |  |  |  |  |
| No | 1 |  |  |  |
| Yes | 0.567 | 0.148 | 2.181 | **0.409** |
| Clavien-Dindo classification Grade Ⅲ |  |  |  |  |
| No | 1 |  |  |  |
| Yes | 0.378 | 0.023 | 6.178 | **0.495** |
| Tumor size |  |  |  |  |
| < 2 cm | 1 |  |  |  |
| ≥ 2cm | 2.338 | 0.464 | 11.785 | **0.304** |

Cl, confidence; HR, hazard ratio; OS, overall survival; CSS, cancer-specific survival; DFS, disease-free survival; IVRFS, Intravesical recurrence free survival

* < 0.05, ** < 0.01

Table supplement 5: Multivariate survival analysis of intravesical recurrence free survival.

| Multivariable analysis | BRFS | | | |
| --- | --- | --- | --- | --- |
|  | HR | (95% CI) | | **P** |
| Surgical approach |  |  |  |  |
| Endoscopic | 1 |  |  |  |
| RNU | 1.790 | 0.796 | 4.022 | **0.159** |
| Sex |  |  |  |  |
| Male | 1 |  |  |  |
| Female | 0.604 | 0.356 | 1.026 | **0.062** |
| Tumor location Renal pelvis |  |  |  |  |
| No | 1 |  |  |  |
| Yes | 1.850 | 1.009 | 3.394 | **0.047** |
| Affected kidney at diagnosis |  |  |  |  |
| Left | 1 |  |  |  |
| Right | 1.973 | 1.116 | 3.489 | **0.019** |
| Bilateral | 0.000 | 0.000 | - | **0.975** |
| Multiplicity |  |  |  |  |
| No | 1 |  |  |  |
| Yes | 1.317 | 0.692 | 2.507 | **0.402** |
| Synchronous bladder tumor |  |  |  |  |
| No | 1 |  |  |  |
| Previous Hx of bladder UC | 5.307 | 2.681 | 10.504 | **<0.001** |
| Concurrent Bladder UC | 2.573 | 1.069 | 6.193 | **0.035** |
| Post-OP urine cytology |  |  |  |  |
| Negative | 1 |  |  |  |
| Atypia | 0.793 | 0.295 | 2.133 | **0.646** |
| Positive | 2.463 | 1.271 | 4.771 | **0.008** |
| Tumor size |  |  |  |  |
| < 2 cm | 1 |  |  |  |
| ≥ 2cm | 0.807 | 0.436 | 1.496 | **0.496** |

Cl, confidence; HR, hazard ratio; OS, overall survival; CSS, cancer-specific survival; DFS, disease-free survival; BRFS, IVRFS, Intravesical recurrence free survival l

* < 0.05, ** < 0.01
